# A Numerical Study on the Effect of Tool Speeds on Temperatures and Material Flow Behaviour in Refill Friction Stir Spot Welding of Thin AA7075-T6 Sheets

**DOI:** 10.3390/ma16083108

**Published:** 2023-04-14

**Authors:** Venkata Somi Reddy Janga, Mokhtar Awang, Srinivasa Rao Pedapati

**Affiliations:** Department of Mechanical Engineering, Universiti Teknologi PETRONAS, Seri Iskandar 32610, Malaysia; venkata_19001587@utp.edu.my (V.S.R.J.); srinivasa.pedapati@utp.edu.my (S.R.P.)

**Keywords:** friction stir spot welding, numerical modeling, refill friction stir spot welding, thermal cycles, simulation of joining processes, material flow, thermomechanical characteristics

## Abstract

A three-dimensional (3D) numerical model was created to simulate and analyze the effect of tool rotational speeds (RS) and plunge rate (PR) on refill friction stir spot welding (refill FSSW) of AA7075-T6 sheets. The numerical model was validated by comparing the temperatures recorded at a subset of locations with those recorded at the exact locations in prior experimental studies from the literature. The peak temperature at the weld center obtained from the numerical model differed by an error of 2.2%. The results showed that with the rise in RS, there was an increase in weld temperatures, effective strains, and time-averaged material flow velocities. With the rise in PR, the temperatures and effective strains were reduced. Material movement in the stir zone (SZ) was improved with the increment of RS. With the rise in PR, the top sheet’s material flow was improved, and the bottom sheet’s material flow was reduced. A deep understanding of the effect of tool RS and PR on refill FSSW joint strength were achieved by correlating the thermal cycles and material flow velocity results obtained from the numerical models to the lap shear strength (LSS) from the literature.

## 1. Introduction

Solid-state joining techniques, friction stir welding (FSW) and friction stir spot welding (FSSW), are used to a great extent for the welding of aluminium and magnesium alloys and are capable of overcoming issues with fusion weldings such as porosity and liquid cracking [1]. In FSW, a rotating tool penetrates perpendicularly (plunging) and then moves transversely in the direction of the weld path. FSW and FSSW are based on the principle of frictional heating. However, FSSW can only plunge, dwell, and retract in an axial direction. The four main categories of FSSW approaches that are now in use are FSSW [2], swept FSSW [3], refill FSSW [4], and swing FSSW [5]. Helmholtz-Zentrum Hereon has developed and patented refill FSSW, a variation of FSSW that produces a spot weld without the exit hole that is unavoidable in FSSW [4].

**Figure 1:**
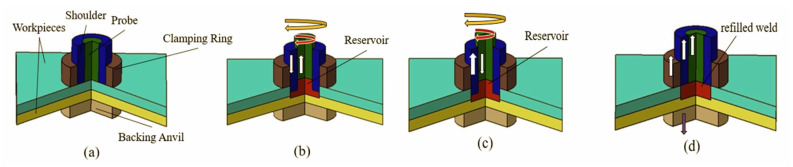
Schematic representation of refill FSSW procedure: (**a**) step 1—initiation; (**b**) step 2—shoulder plunging; (**c**) step 3—refilling; (**d**) step 4—finishing.

Compared to FSSW, refill FSSW offers advantages since it improves weld volume and reduces corrosion cracking, fatigue, and stress concentration [6]. Refill FSSW’s tool assembly comprises a clamping ring, a shoulder, and a probe that move independently. The moving tools, probe, and shoulder plasticize the material through frictional heating. The workpieces are tightly gripped by the clamping ring and a backing anvil, which prevents material from flashing near the tool’s outer edge (shoulder) during the process. The process is classified as probe plunging and shoulder plunging versions subjected to the order of probe/shoulder vertical movement [7]. The shoulder plunging form is the most popular because it results in a more robust, larger-volume weld than the probe plunging type. Figure 1 illustrates the refill FSSW process (shoulder plunging variant).

Step 1: In this step, the clamping force is applied to the workpieces, and the clamping ring and the backing anvil firmly hold the workpieces. The probe and shoulder do not initiate frictional contact with the workpiece and rotate above the workpieces.
Step 2: The rotating shoulder moves vertically downwards and penetrates the workpiece (plunging), and the probes advance vertically in the direction opposite the shoulder. This action initiates deformation and frictional heating. The softened material is drawn into the reservoir; see Figure 1b.
Step 3: The material (plasticized) is refilled/pushed into the workpiece by switching the vertical directions of the rotating tools in this refilling step.
Step 4: The shoulder and probe’s vertical and rotational motions are stopped once Step 3 is finished, and the clamping force is released.

After plunge depth (PD), tool RS is also an essential factor influencing joint strength [8,9,10,11,12,13,14,15,16,17]. According to Shen et al. [8], the increase in weld volume and material mixing are influential in determining joint strength. Previous research on refilling FSSW of aluminium alloys shows that with an increase in RS, joint strength increases initially and then decreases [9,12,14,17,18,19,20,21,22]. Some studies present a decrease [23,24,25,26] and an increase [10,11,27,28] in joint strength with an increase in RS. According to research done by Zhou et al. [20], at lower RSs, voids are formed due to poor material flow, and with an increase in RS, vertical plastic flow is enhanced. Joint strength initially increases and gradually reduces with increased RS due to excessive heat induced by a further rise in RS. Li et al. [23] stated that poor metallurgical bonding exists at the interface of SZ and the thermomechanically affected zone (TMAZ) at lower RSs. Still, there is sufficient stirring at the lap interface. In addition to plasticization, material mixing significantly affects joint quality in friction-stir-based joining processes [29,30]. Increasing the RS increases material flow, enhancing material mixing and the metallurgical bonding at the SZ/TMAZ interface. Still, the dispersed material at the lap interface and coarsening of the microstructure are prone to cracks, reducing the joint strength. Kubit et al. [15] stated that there is insufficient plasticization at lower RSs, and the weakening of the joint is associated with less heat produced due to lower RSs. Overall, from the experimental studies and microstructural observations, it is observed that an increase in RS increases heat input, which induces more plasticity and enhances material flow in the vertical direction. The enhanced material flow improves joint strength and helps avoid defects like voids and incomplete refill in the weld. However, excessive heat input with a further increase in RS will weaken the joint because of the coarsening microstructure and the reduction of bonding ligament length (dispersed lap interface) because of excessive vertical material flow. PR/welding time (WT) is the parameter with the least influence on joint strength [18,22,24,31]. In most of the earlier work, it was observed that joint strength is enhanced with an increase in the WT/decrease in PR [8,10,27,32,33]. The increased WT allows a rise in temperature for proper phase change of the material and improves material flowability and mixing. Similar to the RS trend, in some cases, with an increase in WT, joint strength increases initially and then drops [15,28,31]. In some studies, joint strength decreases with a rise in WT [18,19,24]. The decrease in weld strength is due to the induced excessive temperatures with an increase in WT, similar to increasing RS.

Muci-Küchler et al. [34] used the Abaqus-Explicit code to simulate the probe plunging version of refill FSSW, but the simulation was restricted to the plunging step. The thermal and material flow results were reported, and the model was validated using experimental findings. Ji et al. [35,36] used Ansys Fluent to create a numerical model to examine the material flow velocities in refill FSSW. Since the simulation was conducted using various models at various PDs and presented mainly the material flow velocities, the actual material flow behavior and other quantitative results were not reported. According to their studies, increasing RSs and adding geometric features to the tools improve material flow. They also conclude that increasing the RS is an effective method to enhance material flow. Utilizing DEFORM-3D, Malik et al. [37] refilled the exit hole using multi-stage processes that differ from those of traditional refill FSSW. Quantitative results were not presented despite the exit hole being refilled. Kubit et al. [38] created a two-dimensional (2D) numerical model with Simufact Forming code. The 2D material flow from the simulation was validated with the joint’s microstructures. Zhang et al. [39], using ABAQUS (coupled Eulerian–Lagrangian formulation), developed and validated a 3D thermomechanical model. The material flow characteristics and thermal cycles during refill FSSW of sheets made of magnesium alloy were presented. The numerical models of the processes involving high deformations and temperatures, like friction stir processing, FSW, and FSSW, were developed using DEFORM-3D (Lagrangian incremental formulation) [40,41,42]. The simulations can withstand significant deformations and predict material flow and thermal cycles. A thermomechanical model was created by Janga et al. [43,44] in DEFORM-3D, and the model was validated via temperatures obtained from experiments. The results of the material flow velocities, strain, and temperatures were shown and connected to the experimental findings. Xiaong et al. [45] developed an axisymmetric 2D model to simulate refill FSSW of AA7075-T6 in ForgeNxt3.2 software. The model was validated based on thermal results, and void formation was correlated to material flow. However, as the model was 2D, the outward material shearing under the influence of the rotating tool could not be visualized. Raza et al. [46] explored the evolution of intermetallic compounds propelled by chemical and mechanical forces, considering the effect of several driving forces in a multiphase-field framework numerical model developed in DEFORM-3D.

The RS and PR parameters’ effect in refilling FSSW has been presented in prior experimental studies. However, measuring thermal cycles and understanding the local material flow behavior is challenging, even with micrographs, thermocouples, and start/stop experiments. Also, studying material flow behavior and strain locally experienced in joining thin sheets is quite difficult based on experimental results. In earlier research, a few computational studies demonstrated the material flow and thermal cycles during refill FSSW and correlated the numerical results to the joint characteristics. The thermal cycles and temperatures in the SZ and their influence on refill FSSW joint strength and material flow are still unclear when RS and PR are varied. Furthermore, no numerical investigations presented thermal cycles, strain, and material flow velocities when RS and PR parameters were varied. Therefore, a 3D numerical model was developed and validated in the current study to investigate the thermal cycles and local material flow behavior during the process by varying RSs and PRs.

## 2. Refill FSSW: Finite Element Modeling

The refill FSSW process was modeled in DEFORM-3D using an incremental Lagrangian formulation. The simulations in the current study replicated the prior experimental work done by Yamin et al. [31] for model validation. As a result, the material, tool geometry dimensions, and refill FSSW process parameters were all taken from the experimental investigation. Simulations were run with changing RSs and PRs while keeping all other parameters constant to gain insight into the impact of tool speeds on the process. In the first three models, M1, M2, and M3, RSs of 2000 rpm, 2500 rpm, and 3000 rpm, respectively, were varied, while the PD was 0.7 mm and PR was 0.5 mm/s, unchanged. In the following two simulations, in the models M4 and M5, the PRs were 0.25 mm/s and 0.75 mm/s, while the RS and PD were 3000 rpm and 0.7 mm, respectively.

### 2.1. Refill FSSW Geometry

**Figure 2:**
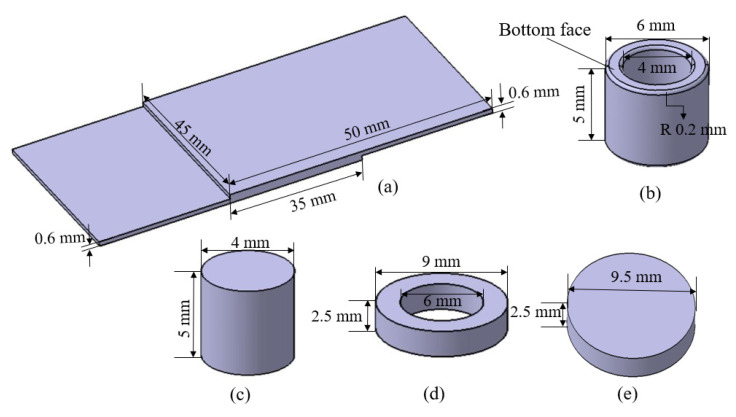
Geometries used for the numerical model with detailed dimensions (**a**) Workpiece; (**b**) Shoulder; (**c**) Probe; (**d**) Clamping Ring; (**e**) Banking Anvil.

A toolset (shoulder, probe, and clamping ring), workpiece, and backing anvil were the geometry utilized in the finite element model of refill FSSW. Figure 2 depicts the detailed dimensions of geometries. CATIA V5R20 was used to model the geometries. A fillet was added for the shoulder bottom edges to reduce penetration stresses during plunging and improve the contact area [34]. The geometries were then assembled in DEFORM-3D. In general, the sheets were firmly clamped. Thus, sheets in the simulation were treated as a combined sheet for simplification. The workpiece’s length and the toolset’s height were restricted to reduce computing time.

### 2.2. Material Law, Materials, and Meshing

It is critical to choose an applicable strain-rate and temperature-dependent law to simulate a material’s response in processes like refill FSSW. In the refill FSSW process and similar operations, the Johnson-Cook material law, which considers the impacts of strain rate, strain, and temperature, is frequently used [44,47]:

(1) $$\sigma_{y}=\left[\;A+B\;{\left[\varepsilon^{pl}\right]}^{n}\;\right]\;\left[1+C\;ln\left(\frac{{\dot{\varepsilon }}^{pl}}{{\dot{\varepsilon }}^{0}}\right)\;\right]\;\left[1-{\left[\frac{T-T_{r}}{T_{m}-T_{r}}\right]}^{m}\right],$$

where $\sigma_{y}$ denotes the material flow stress, the reference plastic strain rate is represented by ${\dot{\varepsilon }}^{0}$, $\varepsilon^{pl}$ denotes the equivalent plastic strain, ${\dot{\varepsilon }}^{pl}$ denotes the plastic strain rate, and $T_{m}$ and $T_{r}$—the melting and reference temperatures, respectively. The material constants n-coefficient of strain hardening, m-thermal softening coefficient, A-quasi-static yield strength, B-strain hardening constant, and the strain-rate dependency are described at the reference strain rate by the strengthening coefficient *C*.

The experiment used a commercial Al-Zn-Mg-Cu alloy AA 7075-T6 with a sheet thickness of 0.6 mm [31]. The material AA7075-T6 applied to the workpiece was loaded from DEFORM-3D’s library and adhered to the Johnson–Cook material law. The Johnson–Cook material law coefficients and constants were taken from a study by Fang et al. [48]. Additional material parameters were Young’s modulus E = 68.9 GPa, thermal conductivity = 180.175 W/m K, Poisson’s ratio = 0.3, specific heat capacity $c_{p}$ = 870 J/kg K, and $\alpha =2.2\times 10^{-5}$/K (thermal expansion coefficient).

Meshing details were as follows—70,000 elements meshed the shoulder and probe, 10,000 elements meshed the clamping ring, 15,000 meshed the backing anvil, and 180,000 meshed the workpiece. Due to the process’ severe deformations, an extensive remeshing scheme was used. A condition connected to the interference depth (0.25 mm) was specified to trigger remeshing. However, remeshing also started when the elements significantly deformed and became unusable.

### 2.3. Boundary Conditions and Contact

For conduction between the workpiece and toolset, a conductive heat transfer coefficient of 11 $\frac{N}{mm\;s\;K}$ was used [43,44]. For convective heat transfer from tools/workpieces to the environment, the heat transfer coefficient h = 0.02 $\frac{N}{mm\;s\;K}$ was used [44,49]. The side faces of the workpiece were constrained in all degrees of freedom. The clamping ring and the backing anvil were made stationary. The rotation and translation movement for the shoulder and probe were defined according to the parameters. Incipient melting might cause tool slippage as temperatures increase. As a result, a friction coefficient that changes as a function of temperature is suggested [50]. Coulomb’s friction law was used in the simulation, with a temperature-dependent friction coefficient [43,44], as shown in Table 1.

The two significant steps in the simulation process were as follows:

1. Plunging stage: The shoulder plunged with the specified PR, RS, and PD. The probe went in the reverse direction vertically, with a speed of 1.25 times that of the shoulder’s PR and the same RS as that of the shoulder. The softened/plasticized material was drawn inwards into the reservoir in this step.
2. Refilling stage: The probe and shoulder switched their vertical movements, maintaining the axial speed of the previous step (plunging step), allowing the material to be refilled.

## 3. Results and Discussion

**Figure 3:**
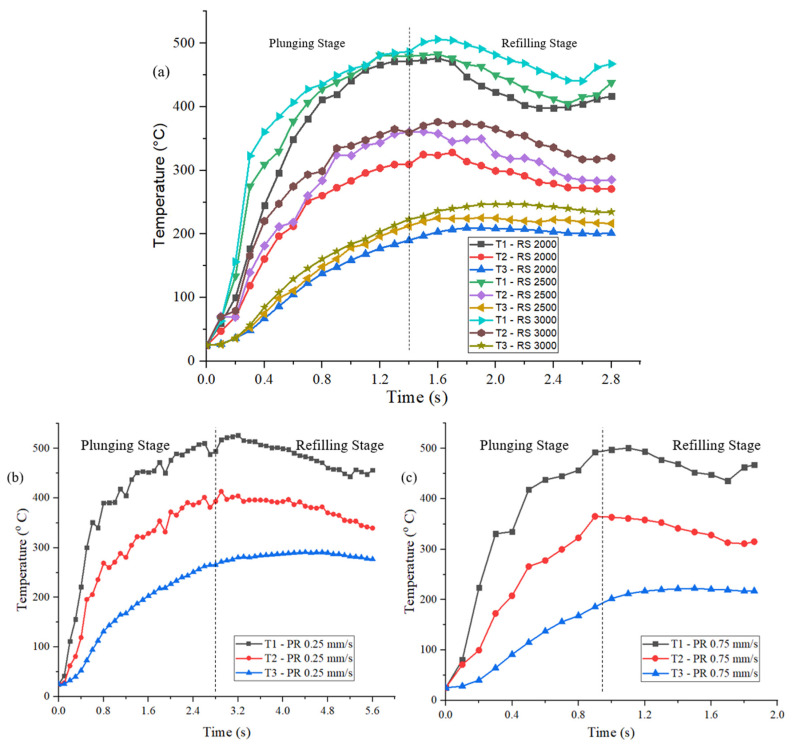
Temperature behavior in the numerical models at characteristic points T1, T2, and T3 of (**a**) models M1, M2, and M3; (**b**) model M4; (**c**) model M5.

The temperatures from the simulations for models M1, M2, and M3, which showed variations of temperatures with RSs, are presented in Figure 3a. These temperature values were recorded at the characteristic points via point tracking at T1, T2, and T3 at the weld center, 4 mm away and 7.5 mm away from the weld center, respectively. The model was validated by correlating model M3 temperature results to experimental temperature findings from previous work [31] at locations T1, T2, and T3. Table 2 compares the maximum temperatures obtained from the simulations with the experimental values. The maximum temperatures at T1, T2, and T3 locations differed by errors of 2.2%, 2.3%, and 6.4%, respectively. There was a strong correlation between numerical and experimental temperature results at all three locations. The temperatures from the simulations for models M4 and M5, which showed variations of temperatures with PRs, are presented in Figure 3b,c. All the models showed a sharp increase in temperature at the start of the plunging stage at measured location T1. The temperature was then steadily increased in the weld zone, after which it gradually decreased as the process ended. The behavior of the temperature curve at location T2 was similar to that at the weld center, although temperatures dropped further than at the center of a weld. A sharp increase seen at T1 was no longer there at T3, and the temperature rose gradually. In all the models, the peak temperatures were shown following the plunge phase and the beginning of the refilling phase, approximately at 0.6 t (t = process time). All the models showed that temperatures dropped further away from the tool’s axis of rotation. Lower temperatures among the RS variation simulations were observed in model M1 because of the lowest RS among the three models. Model M3 had a higher RS than the other models, contributing to its higher temperatures than other models. Among the PR variation simulations from the models M3, M4, and M5, the temperatures at T1, T2, and T3 were higher in model M4 with a 0.25 mm/s PR due to an increased WT. With the increase in PR, the WT and the contact time were reduced; hence, the temperatures were lower in model M3 and model M5 than in model M4. The comparison of the temperatures measured at T1, T2, and T3 from the simulations is tabulated in Table 2.

**Figure 4:**
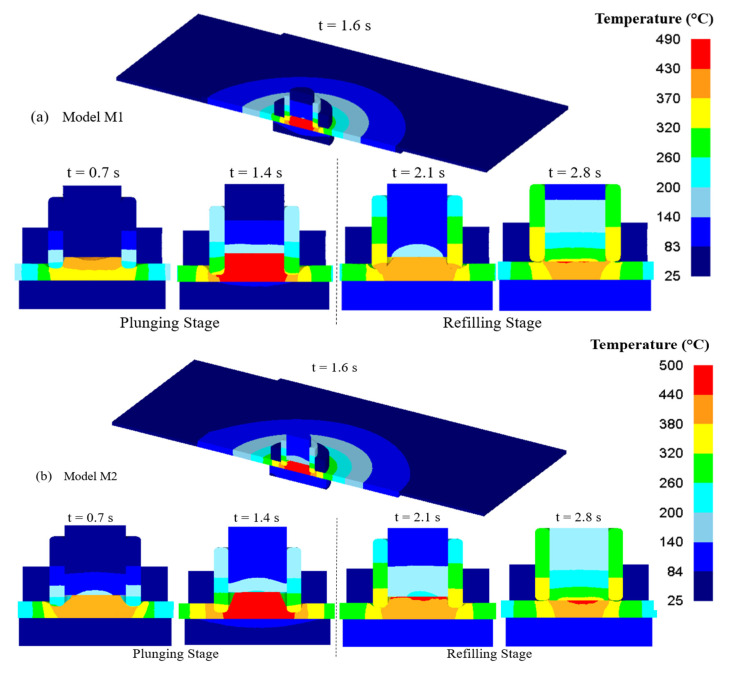

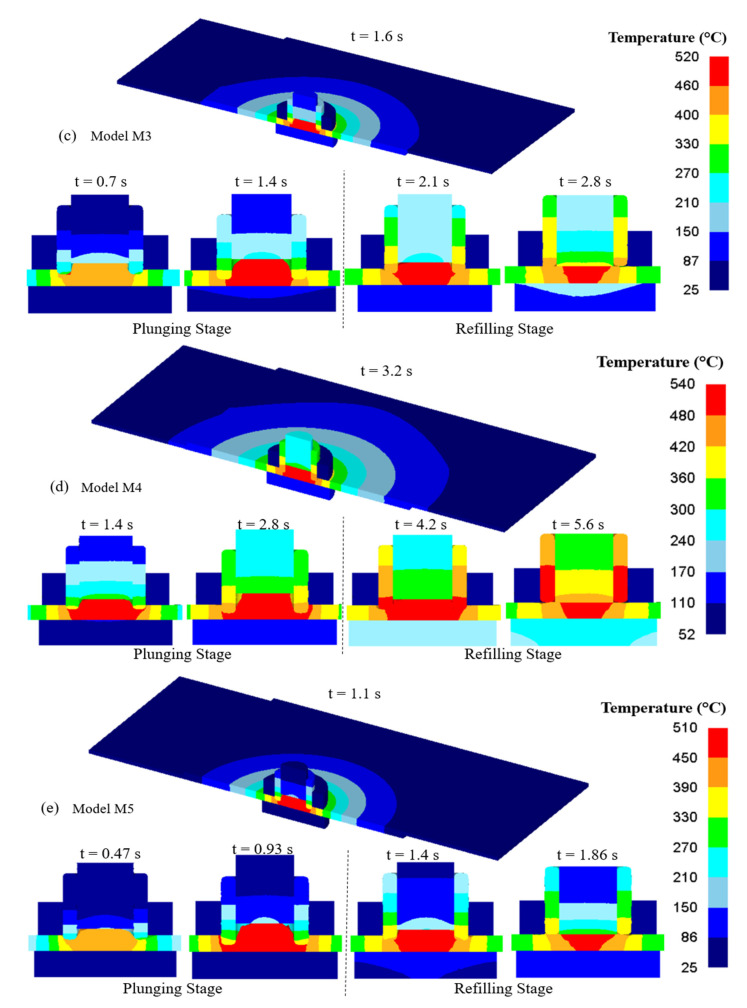
Temperature distribution in the process of refill FSSW from the simulations (**a**) Model M1; (**b**) Model M2; (**c**) Model M3; (**d**) Model M4; (**e**) Model M5.

The temperatures directly impacted the softening and stirring of the material during the process. Figure 4 displays the simulation temperature contours of the three models. The temperatures were distributed symmetrically around the weld center. All models’ temperatures dropped as they moved outward from the weld center. The region where material stirring occurred, or the SZ, was where the highest temperatures were recorded. Models M1, M2, M3, M4, and M5 had maximum temperatures of 490, 500, 520, 540, and 510 °C, respectively, which were 77%, 78%, 81%, 85%, and 80%, respectively, of the material’s melting point (T_m_ = 635 °C). The range of the temperatures in the SZ was from 430 to 490 °C (model M1), from 440 to 500 °C (model M2), from 460 to 520 °C (model M3), from 480 to 540 °C (model M4), and from 450 to 510 °C (model M5). It was evident from the contours that in the SZ, the temperatures were above the solidus temperature (475 °C) [51]. In the range of 475 to 540 °C, the base material’s hardening precipitates (MgZn_2_) dissolve rapidly [52]. As a result, the material in the SZ becomes softer and moves and shears more quickly as the temperatures increase.

**Figure 5:**
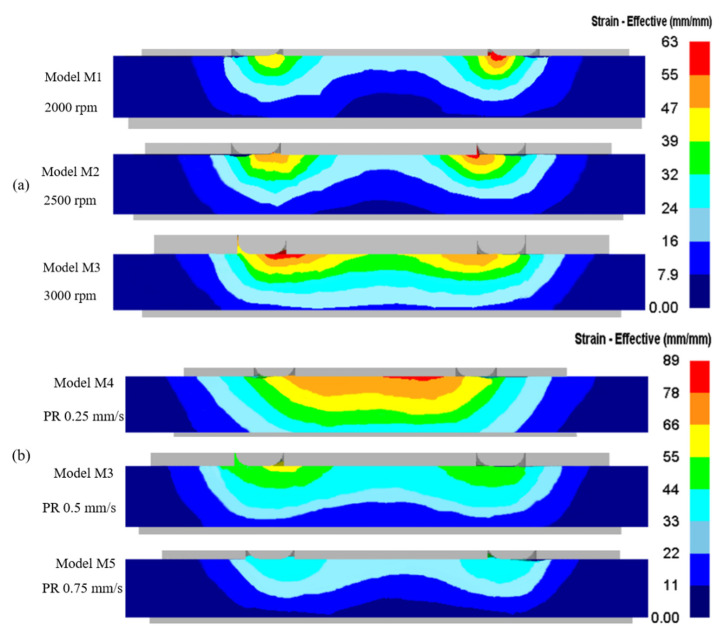
Comparison of effective strain contours at the process’ completion (**a**) at different RSs; (**b**) at different PRs.

The weld’s microstructure and grain size depend on thermal cycles, strain rates, and plastic strains. Figure 5 shows the simulation contours of the effective strain during the process. Generally, microstructure results can be used to identify the distinct zones from the experiments. However, it is challenging to estimate the strain the material experienced locally. All the models had an effective strain contour symmetrically around the weld center. It was clear from the contours that as the RSs increased, effective strain also increased. Dynamic recrystallization, which occurs as a result of extreme plastic deformations and steep temperature gradients in the SZ, produces a finer microstructure [44,53] in the SZ. It was evident from the simulation results that the area of the high-strain zone grew with the rise in RS, indicating more plasticization of the material with an increase in RS [10,15,23]. At lower RSs, sharper and more concentrated effective strain was seen around the shoulder region compared to that at higher RSs. This was because with poor material flow at lower temperatures, the material could stick to the shoulder. However, as the plasticity increased at higher RSs, there was a slip phenomenon consistent with the literature [31]. Experimental observations state that incipient melting occurrs at higher RSs of 2500 and 3000 rpm, which is not seen at the RS of 2000 rpm [31]. The increased RS led to higher temperatures and higher strains, resulting in welding tool slippage in the SZ [31,51,54]. The experimental findings showed that when the temperature reached 495 °C, incipient melting started occurring in the grain boundaries. In contrast, at 480 °C, the sign of incipient melting was not visible. This can also be confirmed by the temperatures obtained from the simulations, see Figure 4. The maximum temperature of model M1 was less than 490 °C; hence, there was no incipient melting and tool slippage, and thus the concentrated, effective strains near the shoulder-affected SZ. A TMAZ adjacent to the SZ has moderate plastic strains and temperatures, due to which there is no recrystallization, and can be identified by elongated grains in the micrographs [44]. The long grains are formed due to the plasticization and tool movement at these moderate temperatures. The TMAZ was extended for a few microns adjacent to the SZ near the shoulder’s outer edge. This range of TMAZ could be identified from the material movement, which will be discussed further. The heat-affected zone (HAZ) refers to the region only impacted by thermal cycles without the material moving mechanically. The weld’s hardness characteristics depend on the thermal cycles and the plastic deformation that the base material undergoes. The condition of isomorphous precipitates that exists after welding becomes the major determinant for hardness because strong plasticization significantly impacts how much precipitates dissolve when stirred [55]. The thermal cycles in HAZ reduce hardness due to coarsening of precipitates. With the temperatures and the plasticization the SZ experiences, the hardening precipitates are dissolved, reducing the hardness and improving the SZ’s capability of hardness by reprecipitation [56]. In the TMAZ, the material becomes the softest as it experiences higher temperatures than the HAZ does, and there is no reprecipitation. As discussed earlier, an increase in RS increases temperatures in the weldments, which affects the hardness of the base material differently in different zones. Therefore, although the increase in RS improves the plasticity/movement of the material, the excessive temperatures that the base material experiences can weaken the joint strength [11,15]. At lower PRs, i.e., with an increase in WT, the effective strains are higher and more distributed than the effective strains from the models with higher PRs; see Figure 5b. As discussed earlier, this was due to increased frictional heating which plasticized the material. Wider distribution of effective strains was observed as WT increased. In model M5, the quickest of the simulations, higher effective strain was seen in a small, concentrated region adjacent to the shoulder. It was observed that PR/WT significantly affected the strain around the weld region.

**Figure 6:**
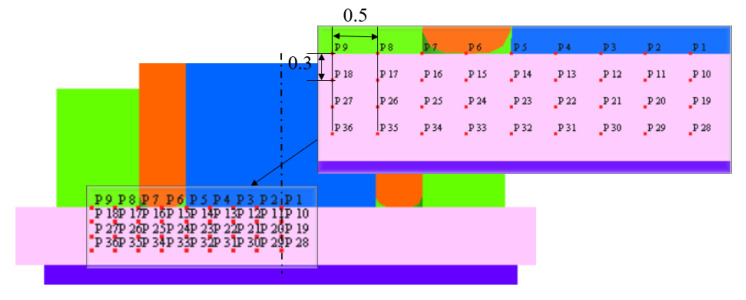
Initial positions of characteristic points using point tracking for material flow analysis.

For a deeper understanding of local material flow behavior with changes in RS and PR, selected points were marked via point tracking, as shown in Figure 6. Considering the process’ symmetrical nature, as observed from the above results, the initial characteristic points via point tracking were marked on the half section; see the zoomed view in Figure 6. The horizontal and vertical distances separating the characteristic points are 0.5 mm and 0.3 mm. Distinct points were P1–P9 (the surface of the workpiece) and P10–P18 (middle) in the top sheet. In the bottom sheet, the characteristic points were P19–P27 (the top of the bottom sheet/interface) and P28–P36 (the middle of the bottom sheet).

**Figure 7:**
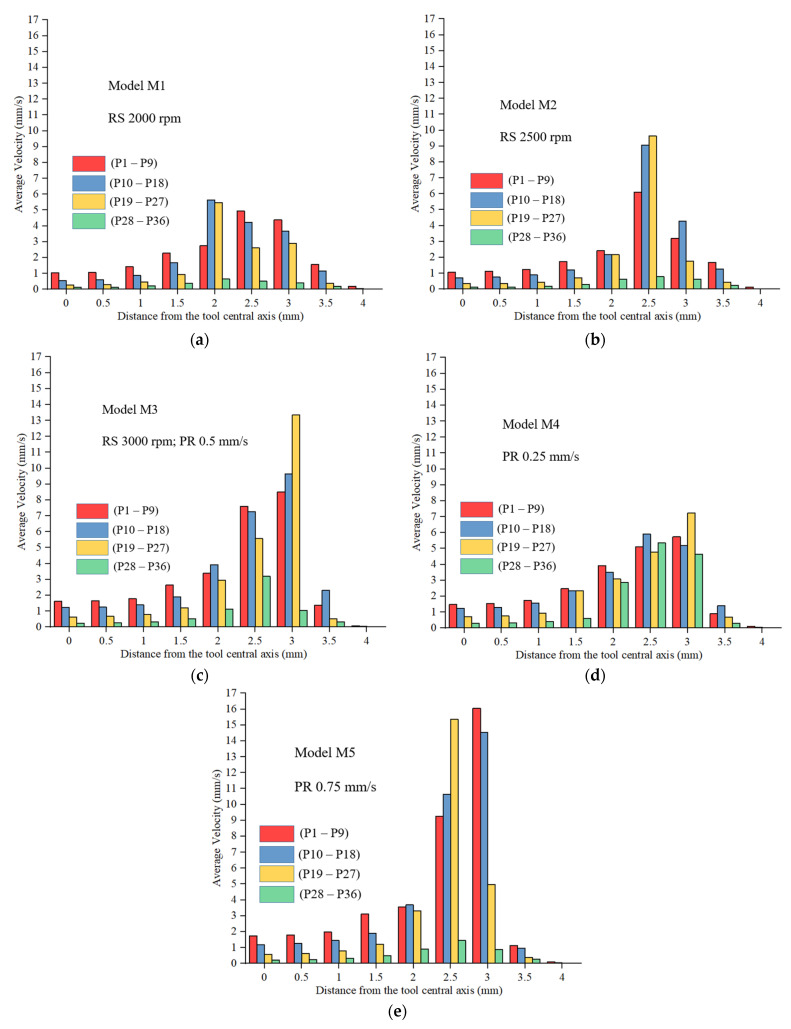
Time-averaged velocities from the characteristic points that are marked in Figure 6, (**a**) model M1; (**b**) model M2; (**c**) model M3; (**d**) model M4; (**e**) model M5.

The material flow patterns with similar parameters of model M3 are discussed in an earlier study by Janga et al. in detail [43]. Therefore, this study does not discuss material flow patterns but focuses on material flow velocities. The time-averaged velocities, derived from the characteristic points in Figure 6 of all the models, are presented in Figure 7. For model M1 with a 2000 rpm RS, the maximum average velocities were detected close to the inner periphery of the shoulder (2 mm from the tool’s rotational axis). The maximum average velocity at this location could be due to the combination of strong outward shearing and inward squeezing according to the material flow patterns observed in a previous study [43]. For model M1, 5.6 mm/s was the maximum average velocity, followed by 5.4 mm/s; related locations were P14 and P23. This further confirmed sticking/no slippage of the shoulder due to lower temperatures, as discussed earlier. The graph shows that more material was squeezed inwards into the reservoir with the increase in RS, as it could be seen that the velocities of the material adjacent to the shoulder’s outer edge were enhanced. The maximum average velocities from models M2 and M3 were seen at 2.5 mm away (material underneath the shoulder) and 3 mm away (material near the outer edge of the shoulder), indicating this phenomenon. The maximum average velocity for model M2 was 9.6 mm/s, and the next was 9 mm/s; related locations P24 and P15. The maximum time-averaged velocity value obtained from model M3 was 13.3 mm/s, and the next was 9.6 mm/s, corresponding to points P25 and P16. The averaged top sheet velocities within the SZ (points P1–P7 and P10–P16) increased by 2.3% and 34.6% from model M1 to model M2 and from model M1 to model M3, respectively. From model M1 to model M2 and model M1 to model M3, the time-averaged velocities from the characteristic points within the SZ in the bottom sheet (points P19–P25 and P28–P34) increased by 15.7% and 51.9%. This shows that the RS significantly affected material flow, and a higher RS improved the flow of materials in the SZ.

For model M4 with 0.25 mm/s PR, the maximum average velocities were seen near the shoulder’s outer periphery. For model M4, 7.2 mm/s was the maximum average velocity, followed by 5.9 mm/s; related locations were P25 and P15. It was observed that material velocities rose as a result of a rise in PRs. The maximum average velocities from models M3 and M5 were seen at 2.5 mm away, i.e., at the material underneath the shoulder, and 3 mm away, i.e., at the material near the outer edge of the shoulder, respectively. A gradual rise of averaged velocities in the shoulder-affected region (between 2 and 3 mm from the central axis) was observed in model M4. The maximum average velocity for model M5 was 16 mm/s, and the next was 15.3 mm/s; related locations P7 and P24. The averaged top sheet velocities within the SZ (points P1–P7 and P10–P16) increased by 19.9% and 40.4% from model M4 to model M3 and from model M4 to model M5, respectively. This shows that PR significantly affected material flow velocities, especially at the material around the shoulder. From model M4 to model M3 and model M4 to model M5, respectively, the time-averaged velocities from the characteristic points within the SZ in the bottom sheet (points P19–P25 and P28–P34) reduced by 6.9% and 8.6%, respectively. This indicates that with the increase in PR, the material moment in the top sheet was enhanced, but the material velocities in the bottom sheet were best with a lower PR. This slight reduction in material velocities in the bottom sheets was due to decreased contact time of the rotating tool and the temperature drop. Additionally, it was evident from the material flow velocity results that there was no significant material movement after 3.5 mm from the rotational axis in all the models, which confirmed significant material movement only in the SZ. As discussed earlier, the TMAZ adjacent to the shoulder’s outer edge was a narrow zone within 3.5 mm from the outer edge of the shoulder.

The lap shear strengths (LSS) reported from the experiments with the same parameters of models M1, M2, M3, M4, and M5 were 2752 N, 2917 N, 3069 N, 2918 N, and 2957 N, respectively [31]. According to the results of the experimental LSS tests, RS had a considerable effect on joint strength after PD, which was the most significant factor. The LSS increased to 5.6% and 10.3% from model M1 to model M2 and from model M1 to model M3, respectively. It was evident from the material flow velocities that the material flow was improved with an increase in RS, hence the increase in LSS [8,20,20,21,23,35]. With the increase in RS, more material was squeezed inwards and involved in stirring and joint formation, particularly the material near the shoulder’s outer periphery and underneath the shoulder. Accordingly, increased material movement was also seen in the bottom sheet with increased RS. Additionally, material flow was aided by the material’s softening as a result of the increasing temperatures in the SZ with the increase in RS. Increased temperatures allow proper phase change and enhance metallurgical bonding between weldments [33,57]. According to the experimental results, the PR parameter had the least influence on joint strength [31]. The LSS slightly increased from model M4 to M3 by 4.9%, and from model M3 to model M5, it was reduced slightly by 3.6%. The lowest LSS in model M4 among the variant of PR’s could be due to excessive temperatures and increased process time. Although the material was softened/plasticized more due to higher temperatures, the weakening of the joint, as discussed earlier, was due to a reduction of hardness in base materials in different zones, especially in the TMAZ, consistent with earlier literature [8,32,44], where minimal hardness was seen at the TMAZ/HAZ interface. In addition, the averaged velocities in the SZ, as discussed earlier, showed the least material movement in model M4. In model M5, material movement was better than in model M4, and the averaged velocities in the SZ were better than in model M3. Still, the contribution to the enhanced material movement in the SZ was due to increased material movement adjacent to the higher-PR shoulder. Overall, the peak temperatures in the SZ, which were close and around 80% of the melting temperature, provided better joint quality (reported in FSW commonly [58]), and 85% and above could be considered excessive temperatures weakening the material. Along with this, material flow was also a key factor for enhancing joint strength. It could be concluded that enhanced material movement and controlled temperatures produced high-quality and high-strength joints.

## 4. Conclusions

The effect of tool RS and PR during refill FSSW of thin AA7075-T6 sheets was studied using a validated 3D thermomechanical model. The thermal cycles were key factors determining the heat input and material softening, which affected material flow and joint quality. It could be concluded that temperatures around 80% of the T_m_ were ideal for joining, and excessive temperatures weakened the joint. In addition to the thermal cycles, material flow in the SZ was another major factor contributing to joint strength. With an increase in RS, the temperatures increased, and more material was squeezed inwards into the stirring, indicating enhanced movement in the SZ, especially adjacent to the shoulder’s outer periphery. The temperatures were reduced with an increase in PR/decrease in WT. Among the PR variants, a more balanced and enhanced material flow was observed in the SZ in the model M3. The LSS of the joint was correlated with material movement and temperature data from the simulations. The numerical analysis provided a deeper insight into the influence of thermal cycles and material flow on joint strength. Overall, enhanced material movement under controlled temperatures leads to superior quality and strong joints.

## Figures and Tables

**Table 1:** Temperature-dependent Coulomb’s friction coefficient used in the numerical model [44].

**Temperature (°C)**	20	160	200	400	500	580
**Coefficient of Friction (µ)**	0.35	0.3	0.26	0.08	0.03	0.01

**Table 2:** Comparison of maximum temperatures from the experiment and simulation.

	Maximum Temperature (°C) at T1	Maximum Temperature (°C) at T2	Maximum Temperature (°C) at T3	Maximum Temperature (°C) in SZ	% of Melting Point (635 °C)
Experiment	495	386	231	-	-
Model M1	476	328	210	490	77
Model M2	483	361	225	500	78
Model M3	506	377	246	520	81
Model M4	527	413	291	540	85
Model M5	501	363	222	510	80

## Data Availability

Not applicable.
